# Cancer medicines: a private vice for public benefit?

**DOI:** 10.3332/ecancer.2024.ed131

**Published:** 2024-01-30

**Authors:** Richard Sullivan

**Affiliations:** Institute of Cancer Policy, Centre for Cancer, Society & Public Health, King’s College London, London SW1 9RT, UK

**Keywords:** affordability, systemic anti cancer therapy, cancer medicines, pharmaceutical policy

## Abstract

Cancer medicines have become one of the most dominant global medical technologies. They generate huge profits for the biopharmaceutical industry as well as fuel the research and advocacy activities of public funders, patient organisations, clinical and scientific communities and entire federal political ecosystems. The mismatch between the price, affordability and value of many cancer medicines and global need has generated significant policy debate, yet we see little change in behaviours from any of the major actors from public research funders through to regulatory authorities. In this policy analysis we examine whether, considering the money and power inherent in this system, any rationale global consensus and policy can be achieved to deliver affordable and equitable cancer medicines that consistently deliver clinically meaningful benefit.

## A bit of history

In 2012, Scannell et al [1] published a seminal paper that was to become required reading in biopharmaceutical sector. At its heart was a diagnosis of why the productivity of the industry was declining. In this he coined the term Eroom’s Law (Moore’s law, backwards). This was the observation that drug discovery was becoming slower and more expensive over time, despite improvements in technology, a trend first observed in the 1980s. The inflation-adjusted cost of developing a new drug roughly doubled every nine years. But in 1990’s this trend was to be dramatically reversed thanks to the start of the molecularly targeted era in cancer medicines. Over night oncology biopharmaceuticals went from being a backwater to the saviour of the sector as a whole.

As Scannell later noted in an interview in 2020, “returns on R&D are stochastic and skewed. Industry makes a disproportionate amount of its profits from a few very big drugs. Even for the big companies, the economics are very sensitive to one or two products, and so long as a few people in the industry are winning, it’s very hard for other people to walk away from the game. So, if some companies in the industry seem to be doing well, firms can plausibly express confidence in their pipeline and scientists, and defend their use of shareholders’ money, even though, on average, they’re going to lose some of it.”

Why is this piece of history so important? Because it set in motion a series of global trends that leads us to where we are today. And goes some way to explaining why, despite overwhelming evidence that the current ecosystems governing research and market access for systemic anti cancer drugs (SACT) are not affordable, equitable or sustainable, little appears to be changing. Ten years on from the first ever Lancet Oncology Commission on Affordable Cancer Care in High Income Countries [2] all countries are struggling to address a sustainable model for affordable SACT as well as the costs of waves of new and expensive technologies in radiotherapy, surgery, imaging, *etc*.

## Cancer biopharmaceuticals is fundamentally about making money

This headline statement upsets people. It upsets them because we don’t like to face reality. And that reality is that the biopharmaceuticals industry is about making a profit for shareholders. And profit does not, *per se*, have a limit. The engine for this is the USA which is, globally, the main actor when it comes to incentivising oncology biopharmaceutical R&D. And it is the US legislative ecosystem that allows these companies infinite pricing power. A power that has global implications. Put bluntly the cancer R&D system is over incentivised and this leads to marginal drugs.

One of the most detailed analysis around the costs of R&D and returns on investment illuminates just how strong the fiscal currents are when it comes to oncology. It’s worth noting that in this analysis none of the newer immuno-oncology drugs were included because it it is too early to assess the costs of their R&D and ROI. However, the analysis included the most recent tranche of targeted cancer medicines drawing on detailed financial data filed with the US Securities and Exchange Commission (SEC) [3].

Over 23 years of cancer medicine R&D the mean cost in USD of developing a cancer medicine to pharmaceutical companies has been around $4.4 billion. But this hides a huge range of costs from ‘just’ 276 million USD for Dinutuximab for post consolidation therapy for childhood high risk neuroblastoma to 13.4 and 15.8 billion USD, respectively, for Durvalumab (used to treat certain types of bladder, lung, and biliary tract cancer) and Isatuximab (used in treatment of blood cancer multiple myeloma).

This reflects the fact that the true costs of R&D cover a very wide range, and these expenditures do not, and never have had have any relationship to downstream prices in any market. Moreover none of these figures take into account public underpinning R&D funding which is intimately woven into the private sector [4]. The price of SACT has drawn in huge amounts of R&D capital which means ever increasing competition in shrinking therapeutic spaces, duplication and low societal returns.

The data on returns on investments is even more startling. The first point to make is that bringing any cancer medicine to market generates substantial returns to the company(s), irrespective of of the return on investment Even bringing a cancer medicine along the pipeline ensures substantial capital flows in terms of inward R&D investment.

Overall, ROI for cancer medicines with sufficient maturity i.e., launched between 1997-2015 is between 435 to 551%. Again, this hides huge variation. A substantial number of cancer medicines to date have negative or flat ROI’s as low as minus 78-87% in some cases. However, some cancer medicines launched in late 1990’s to mid 2000 have generated astronomical ROIs; for example, Erlotinib (2794%) (pancreatic and lung cancer), Trastuzumab (3421%) (breast cancer), Rituximab (2523%) (Non Hodgkin’s Lymphoma) and Bevacizumab (3200%) (colon, lung, glioblastoma and renal cell cancers). This reflects the fact the the oncology business model is still driven by blockbusters.

Whilst this work provides more clarity based on real figures filed with US SEC it also shines a light on the vast financial complexity of the biopharmaceutical industry. Indeed, we know that the ROI are almost certainly higher than the figures calculated and that these figures continue to increase and expand year-on year. There is compelling evidence that the precision medicines companion diagnostic driven cancer drug development is likely to reduce R&D costs, however, there is no evidence that this will flow into lower downstream prices. If anything, premium pricing, ‘what the market will bear’, is driving up prices and costs of cancer medicines across all ecosystems and this will continue.

We know from work of Lazonick [5] and others that, contrary to prevailing narrative, the majority of pharmaceutical industry profits are not ploughed back into R&D but instead used for open market stock buy-backs. One of the main mechanisms for keeping stocks high. These stock buybacks, which are in addition to dividend payments, are done in the name of “maximizing shareholder value” (MSV). Buybacks give manipulative boosts to the company’s stock price and, by reducing the number of shares outstanding, increase earnings per share (EPS), a widely accepted indicator of a company’s performance. Rising stock prices enrich senior executives, who receive most of their compensation in the forms of stock options and stock awards [6].

Why is there such a drive to push through new cancer medicines that have such low or non existent clinically meaningful benefit profiles? The answer to a degree rest with the power of incentivisation to drive capital into both biotechnology and pharmaceutical sectors. Through such speculation, it is quite possible for investors to reap huge rewards by trading in biotech and pharmaceutical stocks, irrespective of the benefit it delivers.

Pisano [7] argued that “only approximately 20% of all publicly held companies in existence today have any products on the market or are earning royalties based on products commercialized by partners. Thus, the vast majority of publicly held biotech ﬁrms working in oncology are essentially R&D entities.” The problem thus is that the entire structure and incentive framework governing the biotechnology and pharmaceutical industry are geared towards a specific type of behaviour. Short of completely reforming the entire capital-industrial market, starting in the USA, expecting industry to behave any differently from what it is doing right now is a dead end. It is an ideological and technical *cul-de-sac*. Arguing about the rights and wrongs of industry profits in oncology misses the point. The system is geared towards profit maximisation that is completely independent of R&D costs, it is insensitive to whether the drugs deliver meaningful therapeutic benefit, or whether the cancer medicines are priced ‘fairly’ for any given country.

## The private capture of the public

SACT dominates global oncology R&D. The private sector has captured all major avenues of pre-clinical and clinical cancer drug development, including RCTs [8]. In so doing the private sector are now in pole position to set the research agenda as well as the design of clinical studies. In that respect it hardly comes as a surprise that surrogate endpoints that ensure the fastest possible path through clinical development to market, whether valid or not, have dominated. Manipulation of data has become normalised within the cancer medicines ecosystem; overplaying efficacy and downplaying toxicity and issues of tolerability. In the former the issue of unvalidated or partially validated endpoints have been loudly articulated, yet this has had little impact on either clinical trial design or regulatory approval [9, 10]. Whitewashing of serious toxicity has also become a norm. The development of the mTor inhibitor everolimus is a particularly egregious case of downplaying toxicity [11]. Meaningful progress in cancer drug development requires, as Labadie and Fojo [12] has said, ‘*rigorous interpretation of data and balanced clinical trial designs. It also requires strategies that limit informative censoring in outcomes assessment to at most a few percent lest we declare them uninterpretable’*. But these systemic failures are waved away with the notion that such evidential lacunae can be filled. The assumption has been that it does not matter that some SACT make it into clinical ecosystem early with such huge uncertainty as to their clinical and economic value because post marketing research will separate the wheat from the chaff. This is, supposedly, where Real World Evidence (RWE) comes to the rescue. The reality is somewhat different with shocking deficits in the quality and range of RWE that makes most studies unfit to guide clinical practise or policy [13, 14].

The consequences of an R&D ecosystem constructed around the private sector find common cause in the culture of profit. Much of this is driven by the culture of R&D from USA. As Sahlins [15] put it, “*the systematic organization of the research university by bourgeois subjectivity and entrepreneurial activity or, in other words, the pursuit of disinterested knowledge by self-interested people*”. There is no contradiction between private profit and public good in this model. Higher institutions – universities and major cancer centres - acts as as sub-contractor of the bioscience economy. The public institutions and all public money serve society by also serving the private sector, to which society owes its wealth, knowledge, and way of life. In almost every analysis of research publications SACT (and associated research domains) dominate research outputs whether one looks at USA oncology R&D ecosystem or more socialised systems across Europe [16]. Major public research funding organisations are also party to this capture. Their research portfolios heavily geared towards private sector aligned biopharmaceutical R&D. This domination is total. And high-income countries are not alone. Major emerging economies such as China are also hugely invested in SACT, particularly in next generation biologicals / immuno-oncology [17]. Nascent R&D systems, such as those across MENA [18] and Sub Saharan Africa [19] are also finding common cause in the same biopharmaceutical domains. This begs the questions as to what now constitutes true public good oncology research. Public research funding organisations strategies have become, essentially, pharmaceuticalised [20]. This is not just private sector profit, which in itself is completely normative, rather it is also about the reliance and addiction of countries, patient advocacy groups, public research funders, universities as well as the clinical and scientific communities to the substantial flow of private sector funding that comes with strategic alignment to the biopharmaceutical industries.

## Does anyone actually care?

Let’s start with profit. From 2000 to 2018, 35 large pharmaceutical companies reported cumulative revenue of $11.5 trillion with a gross profit of $8.6 trillion [21]. These are staggering and oncology is one of the major contributors to these figures [22]. Whilst there has been a fair amount of dialogue around the special obligations of the pharmaceutical industry during emergencies e.g., pandemics when it comes to pricing the debate around cancer medicines has been sparse and uneven [23]. Generally arguments that industry has to balance their responsibilities to patients with the profit expectations of their stakeholders has found little fertile ground where the profitability of large pharmaceutical companies is significant [21]. By and large no public research funders, government’s, cancer patient organisations or professional organisations like ASCO or ESMO have ever called for profit restraint, or even proportional (i.e. fair) pricing.

The consensus is that pricing of SACT in most countries without significant governance is unfair and the impact on patients is, in most systems, ruinous [24]. They exceed affordability and value for money thresholds and are not justified by research and development costs [25]. To be fair to industry there is, in many countries, also staggering rent seeking behaviour between the manufacturer and the patient. In many countries multiple actors, including hospitals and doctors, take their ‘cut’ along the supply chain [26]. There is very little discussion about this. But what this means is that the the problem set is systemic. If you care about unaffordable prices then it’s not enough to simply tackle industry, you must tackle the entire profit chain.

Part of the price problem is also due to ‘wasted’ R&D. For example, program failures around the development of targeted inhibitors of the insulin-like growth factor-1 receptor (IGF-1R) may have cost in the region of $1.6 billion USD. Such failures reflect avoidable waste in terms of both time, money, and patients. For a variety of reasons, these ‘journeys to failure’ leave no lesson learnt [27]. And the system is geared towards repeating these costly mistakes. Downstream these failures, and costs, are woven into higher prices. Beyond these pipeline losses the system is also increasingly geared towards allowing market access to new SACT (or new indications) that, by any measure, do not deliver clinically meaningful benefit. Yet so promiscuous are our regulatory systems and so conflicted are the communities championing all new SACT (or indications) that the worthwhile and the useless are mixed with equal applause. By and large no one raises any objections. Patient organisations, public research funding organisations, professional bodies, rarely raise a hint of criticism. And of course, the problem then is that public funds are spent both on meaningful, worthwhile new SACT and the, frankly, worthless. And this has serious cost opportunities. There are some outstanding new types of SACT (and new indications) but these are crowded out with a whole slew of that do not deliver clinically meaningful benefit.

Why the respective cancer communities seem to care so little about this is, to some extent, a measure of how deep the conflicts of interest run. There is a staggering reliance on the pharma dollar within almost every quarter of oncology. Patient organisations exist because of private sector funding, scientific and clinical careers are made by leveraging large pharmaceutical grants, overheads from these grants prop up university and hospital systems, involvement in novel or larger scale pharmaceutical trials brings power and esteem, and professional bodies can expand and thrive entirely because of the substantial private sector funding. It’s difficult to care about the downstream consequences of poor R&D and weak regulations on prices when you are making so much money. In countries such as the USA direct payments to medical oncologists who set national guidelines are now the norm [28]. The USA is not alone with significant transparency problems even in socialised countries such as the UK [29]. Significant increases in *ad hominum* payments hide a bigger issue. The fact is that there are multi-million funding flows into hospitals and universities with little to no scrutiny as to whether the trials or research reflect either an important question or a well-designed study. Indeed career advancement and esteem are almost entirely due to how much money one brings in, not whether the research in question is of any value [30].

Ultimately it is for regulatory authorities – FDA, EMA *etc* – to act as arbiters of public good where medicines are concerned. But judged by the yardstick as to whether new SACT or new indications are consistently providing clinically meaningful benefit, regulation has failed. Half of the RCT that were used to support EMA approval between 2014 and 2016 were judged to be at high risk of bias based on their design, conduct, or analysis. And subsequent journal publications did not reflect key limitations of the available evidence identified in regulatory documents [31]. Too many new SACT or new indications gain regulatory approval without clear demonstration as to their benefit (improving overall survival and / or quality of life) [32]. Only a third of FDA approvals of therapeutic agents for use in solid tumour oncology for the period 2017-2021 were judged to deliver significant clinically meaningful benefit [33]. But, apart from a raft of op-eds and analytical publications this extraordinary imbalance has generated not a single policy ripple. The behaviours of regulatory authorities remain untouched, and authorisations have, if anything, gathered pace as the mantra of ‘fast to market’ becomes the political standard for delivering patient benefit [34, 35].

## Power and money

The root cause of the problem of SACT rests squarely with the USA. Its over incentivisation of cancer biopharmaceutical R&D, it’s ability to provide industry with infinite power when it comes to setting prices, the completely interwoven public and private systems (thanks to the 1980 Bayh-Dole Act), and the huge global impact of the FDA through initiatives such as Project Orbis have set the power and money agenda.

In seeking to find solutions we have witnessed a veritable cornucopia of publications and initiatives in the last decade seeking to illuminate and address the interlocking problem set. Entire movements seeking to redress the balance have been created. The WHO’s Essential Medicine List for Cancer committee has absorbed pricing and afforability as key aspects of its deliberations [36], Choosing Wisely initiatives have taken aim at over-pricing [37], national initiatives such as India’s National cancer Grid have sought to set fairer prices through pooled procurement [38], and movements like Common Sense Oncology [39] have been set up to try and more widely promote issues in poor trial design. In that sense there is greater awareness and sensitisation around the issues than ever before. Some global organisations, Clinton Health Access Initiative for example, have practically championed better and more affordable cancer medicines access. Some countries have also worked hard to try and manage their SACT ecosystem on both demand and supply side. And compared to 20 years ago we are immeasurably better off in terms of more effective SACT for a whole range of cancers.

But stand back for a moment and ask what has really changed? Has the narrative improved? Do we have more balanced realistic discussions? Are prices fairer and is, globally, SACT regimens now more affordable? Not a bit. If anything the hype and hyperbole has just worsened [40]. The inherent power that this much money brings has captured swathes of territory; public research funders, regulatory authorities, and even entire regions [41]. We can write about how commercial determinants are unbalancing services and systems until we are blue in the face [42], but, like the Greek chorus, the words have no impact on the main actors. There is not a shred of evidence that, taken as a whole, the SACT ecosystem is changing course. The oncology pipelines continue to expand, regulatory authorities continue to reduce evidential requirements for market entry, patient and professional bodies continue to loudly cheer each and every new cancer drug, irrespective of price or real clinical benefit, and policymakers fall further behind in their ability to deliver a governance system. And when the going gets tough i.e., some random cabal of interest groups and industry utilise their money and power to play politics then governments routinely cave in. One of the most egregious examples of this being the creation of the Cancer Drugs Fund by the UK [43].

## A sort of conclusion

One is faced with two choices. Accept that progress is a private vice with public benefit. Essentially align with Bernard Mandeville’s position in Fable of the Bees (1705) that vicious greed, properly channelled by skilful politics, will lead to invisible co-operation and public benefit. Espousing any higher virtue is mere hypocrisy. In this world the only challenge is external. If, for example, China was to exercise its considerable biopharmaceutical muscle in oncology to massively undercut global prices. The other choice falls more in line with the Rawlsian idea of social justice. In this world a new social contract is constructed that truly reflects equitable value. Respective institutions all align along this common public good backbone. Prices truly reflect clinical benefit and are set to a fair level that maximises patient access. R&D is incentivised on societal worth and not profit.

For this author the latter position is emotionally the more gratifying. But is it *realpolitik*? The reality of power and money manifested through contemporary politics means that SACT will continue to be dominated and directed by private interests until a crisis forces a radical change. Waiting for societies critical institutions to honestly reflect the true magnitude of benefits as well as the yawning affordability gap might well be likened to waiting for Hell to freeze over [44]. Whilst we remain addicted to the money and prestige nothing will change. But ideas throughout history have always had the power to move the unmovable. And revolutions can come from the most unlikely quarters. What may finally derail the situation we have today may have nothing to do with an direct change of oncology policy and practise. More than likely it will be, as the historian Niall Ferguson discusses in the Great Degeneration (2013), a confluence of externalities that simply stop people and countries funding the system in the way we do now and paying high prices for poor value.

## Funding

RS is supported by Medical Research Council Grant GACD-025.

## Conflict of interest statement

RS has no conflicts of intertest to declare.
